# Reproduction of the Antitumor Effect of Cisplatin and Cetuximab Using a Three-dimensional Spheroid Model in Oral Cancer

**DOI:** 10.7150/ijms.74109

**Published:** 2022-07-18

**Authors:** Kisho Ono, Kohei Sato, Tomoya Nakamura, Yume Yoshida, Shogo Murata, Kunihiro Yoshida, Hideka Kanemoto, Koki Umemori, Hotaka Kawai, Kyoichi Obata, Shoji Ryumon, Kazuaki Hasegawa, Yuki Kunisada, Tatsuo Okui, Soichiro Ibaragi, Hitoshi Nagatsuka, Akira Sasaki

**Affiliations:** 1 Department of Oral and Maxillofacial Surgery, Graduate School of Medicine, Dentistry and Pharmaceutical Sciences, Okayama University, Okayama 700-8525, Japan.; 2 Department of Oral Pathology and Medicine, Graduate School of Medicine, Dentistry and Pharmaceutical Sciences, Okayama University, Okayama 700-8525, Japan.; 3 Department of Oral and Maxillofacial Surgery, Shimane University Faculty of Medicine, Izumo, Shimane 693-8501, Japan.

**Keywords:** oral cancer, spheroid, three-dimensional culture, anticancer drug

## Abstract

**Background/Aim:** Cancer research has been conducted using cultured cells as part of drug discovery testing, but conventional two-dimensional culture methods are unable to reflect the complex tumor microenvironment. On the other hand, three-dimensional cultures have recently been attracting attention as *in vitro* models that more closely resemble the *in vivo* physiological environment. The purpose of this study was to establish a 3D culture method for oral cancer and to verify its practicality.

**Materials and Methods:** Three-dimensional cultures were performed using several oral cancer cell lines. Western blotting was used for protein expression analysis of the collected cell masses (spheroids), and H-E staining was used for structural observation. The cultures were exposed to cisplatin and cetuximab and the morphological changes of spheroids over time and the expression changes of target proteins were compared.

**Results:** Each cell line formed spheroidal cell aggregates and showed enhancement of cell adhesion molecules over time. H-E staining showed tumor tissue-like structures specific to each cell line. Cisplatin showed concentration-dependent antitumor effects due to loss of cell adhesion and spheroid disruption in each cell line, while cetuximab exhibited antitumor effects that correlated with EGFR expression in each cell line.

**Conclusion:** Spheroids made from oral cancer cell lines appeared to have tumor-like characteristics that may reflect their clinical significance. In the future, it may become possible to produce tumor spheroids from tissue samples of oral cancer patients, and then apply them to drug screening and to develop individualized diagnostic and treatment methods.

## Introduction

A commonly used *in vitro* experimental method in cancer research is two-dimensional (2D)-culture using dishes or flasks. However, the cellular environment under 2D culture is very different and often a poor reflection of the complex tumor microenvironment (TME) *in vivo* 1. In 2D culture, all cells can proliferate at a relatively constant rate in a planar space, and can be provided uniform access to nutrients, oxygen or drugs. Furthermore, it is presumed that most of the characteristics inherent in cancer cells are lost. Therefore, it is difficult to propose that 2D culture accurately reflects the original growth state of tumor tissue.

In recent years, a spheroid model produced by three-dimensional (3D)-culture has attracted attention as a better experimental model that is closer to the physiological environment *in vivo* 2. The intra- and extra-cellular events that occur in 3D culture are similar to those in a physiological environment 3-5. Specifically, it is possible to create site-specific differences in the cell proliferation rate and cell morphology/function, imitate microenvironmental conditions such as hypoxia and particular nutritional gradients, and predict the biological response to drug treatment 6. This is clinically important in analyzing the characteristics of intractable cancers, such as cancer treatment resistance and tumorigenicity.

Cisplatin is the most widely used cell-killing anticancer drug for the treatment of oral cancer 7, 8. It induces anticancer effects by causing cellular DNA damage and replication defects, transcriptional inhibition, and cell cycle arrest, resulting in apoptosis. On the other hand, cetuximab, an EGFR inhibitor, is the only molecular targeting agent currently available for head and neck cancer 9, 10. Cetuximab exerts an antitumor effect by competitively binding to EGFR on the cell surface, thereby inhibiting the function of EGFR and blocking the signal transduction that mediates cell proliferation. The efficacy and mechanism of action of these and other anticancer drugs should be properly reproduced and evaluated *in vitro* to clarify their antitumor effects.

In the present study, we aimed to establish a 3D-culture model using oral cancer cells, and attempted to create a more useful *in vitro* disease model. In addition, in order to verify the practicality of this model, we compared the utility of 2D- and 3D-culture models for analyzing the response of different oral cancer cell lines to cisplatin and cetuximab.

## Materials and methods

### Cell lines and 2D cell culture

The human oral squamous cell carcinoma lines SAS, HSC-3, HSC-4, and OSC-19 were obtained from JCRB Cell Bank. All of these cell lines were cultured in Dulbecco's modified Eagle's medium (DMEM) (Thermo Fisher Scientific, Waltham, MA) supplemented with 10% heat-inactivated fetal bovine serum (FBS) and 1% penicillin-streptomycin. They were cultured in an atmosphere of 5% CO2 at 37 °C.

### Reagents

Antibodies against EGFR (D38B1, #4267S), p-EGFR (D7A5, #3777S), E-cadherin (24E10, #3195S), cleaved caspase-3 (D175, 5A1E, #9664S), caspase-3 (8G10, #9665), GAPDH (D16H11, #8884S) and Ki-67 (D2H10, #9027S) were purchased from Cell Signaling Technology (Danvers, MA). HRP-conjugated anti-β-actin antibody (ab49900) was purchased from Abcam (Cambridge, MA).

### Chemicals and drugs

Cisplatin (Randa® Injection) was obtained from Nippon Kayaku (Tokyo) and Cetuximab (Erbitux® Injection) was from MerckSerono (Tokyo).

### 3D spheroid culture and morphological analysis

3D spheroids were cultured in 96-well ultra-low attachment (ULA) plate (#7007; Corning, NY). 1.0×10^5^ cells were seeded in each well and cultured in the same environment as described above for the 2D culture. The same seeding methods were used for all four oral cancer cell lines. Medium was changed every three days and maintained at a volume of 100 µl/well. However, to avoid accidental aspiration of spheroids, only half the total volume of medium was exchanged on each occasion. Spheroid microplates were placed in a humidified incubator set at 37 °C and 5% CO2. Images of spheroids were captured using a bright-field microscope (IX81; Olympus, Tokyo).

### Drug treatment

Cisplatin was added to the formed spheroids in a stepwise concentration (0, 1, 2 μg/ml) 24 h after cell seeding to the ULA plate (Fig. 3A). Cetuximab was also added to the formed spheroids in a stepwise concentration (0, 60, 90 nM) 24 h after cell seeding to the ULA plate (Fig. 4A). An appropriate amount of each drug was added to maintain the concentration during the medium changes every three days.

For 2D culture, cells were seeded in a 96-well 2D-culture plate in DMEM supplemented with 10% FBS for 24 h, and then the drugs were added and the cells were cultured for 48 h.

### Cell viability assay

Cisplatin was added to the oral cancer cells under 2D and 3D culture in a half maximal inhibitory concentration (IC50) 24 h after cell seeding. PBS was added to the control group as a substitute for cisplatin. Cells and spheroids were detached or disassembled using Trypsin/EDTA at 24 h after the addition of cisplatin or PBS, and the number of cells was counted using a Countess® Automated Cell Counter (ThermoFisher Scientific). Cell viability was determined by converting the ratio of the number of cells in the cisplatin- and PBS-added groups. An MTT assay was performed to determine the IC50 of cisplatin on each oral cancer cell line under 2D culture.

### Whole cell lysate

The whole cell lysate (WCL) was prepared as described previously 11. Briefly, cells cultured in a 10-cm dish were lysed in 150-200 μL/dish of RIPA buffer (1% NP-40, 0.1% SDS, and 0.5% deoxycholate, and EDTA-free protease inhibitor cocktail in PBS) and collected by using a cell scraper. Cells were further lysed using a 25-gauge syringe for 10 strokes and then incubated for 30 min on ice. 3D spheroids were treated with ultrasonic crushing after lysing in RIPA buffer. The protein concentration of the WCL was analyzed using a BCA protein assay (Thermo Fisher Scientific).

### Western blotting (WB)

WB was performed as described previously 11. The same protein amounts of WCL were subjected to SDS-PAGE, followed by transfer to a PVDF membrane using semi-dry methods where appropriate. The membranes were blocked in 5% skim milk in Tris-buffered saline containing 0.05% Tween 20 for 60 min, incubated with primary antibodies, and then incubated with horseradish peroxidase (HRP)-conjugated secondary antibodies. Blots were visualized according to the Clarity ECL protocol (Bio-Rad, Hercules, CA). A ChemiDoc MP system (Bio-Rad) was used for analysis of the WB.

### Preparation of spheroid sections

Pipetting or aspiration was used to remove as much media as possible without disturbing the spheroids. Spheroids were washed with PBS and fixed in 4% PFA for 30 min. Spheroids were additionally washed with PBS three times for 5 min each time and embedded in paraffin. Spheroid sections (3 µm thickness) were deparaffinized and hydrated through xylenes and a graded alcohol series. Antigen retrieval was performed by dunking in proteinase K for 5 min. Sections were additionally washed with Wash Buffer (Dako, Carpenteria, CA, USA) two times for 5 min each time, and then endogenous peroxidase activity was blocked by treatment with blocking solution (Dako) for 30 min at RT, and the sections were incubated with primary antibodies.

### Immunohistochemistry (IHC)

We analyzed the expression of Ki-67 and cleaved caspase-3 in 3D spheroids by immunohistochemical analysis. The sections were incubated with anti-Ki-67 antibody (dilution 1:200) or anti-cleaved caspase-3 antibody (dilution 1:1000) overnight at 4 °C, followed by treatment with avidin-biotin complex (1:100, VECTASTEIN Elite ABC Kit; Vector Labs, Burlingame, CA, USA) for 60 min, and visualized with the use of a DAB substrate-chromogen solution (DAB Peroxidase Substrate Kit, ImmPACT; Vector Labs). The staining results were observed with an optical microscope BX53 (Olympus).

For 2D IHC, cells were cultured on coverslips coated with collagen and fixed in 4% paraformaldehyde in PBS for 10 min at RT. Fixed cells were permeabilized for 10 min in PBS containing 0.1% Triton X-100. Endogenous peroxidases were blocked for 30 min in 1.5% H2O2 for 30 min. For blocking non-specific reactions of primary antibodies, cells were incubated for 30 min at RT in 1% bovine serum albumin (BSA) in PBS containing 0.1% Tween 20. The applied primary antibodies were follows: anti-E-cadherin antibody (dilution 1:100) and anti-EGFR antibody (dilution 1:100). The process from immunostaining to imaging is the same as above.

### Hematoxylin and eosin (H-E) staining

Spheroids sections were deparaffinized in a series of xylene for 15 min, rehydrated in graded ethanol solutions, and washed well in distilled water. Then sections were incubated in Mayer's hematoxylin solution for 7 min and rinsed in tap water until the water was colorless. Finally, after sequential treatment with hydrogen chloride and 80% ethanol solution, sections were incubated in eosin for 7 min.

### Statistical analysis

Statistical significance was calculated using Microsoft Excel. Differences between two sets of data were examined with a paired Student's t-test; values of p<0.05 were considered to indicate statistical significance. Data were expressed as means ± S.D. unless otherwise specified.

## Results

### Morphological observation of oral cancer cell lines in 2D and 3D culture

**Figure 1:**
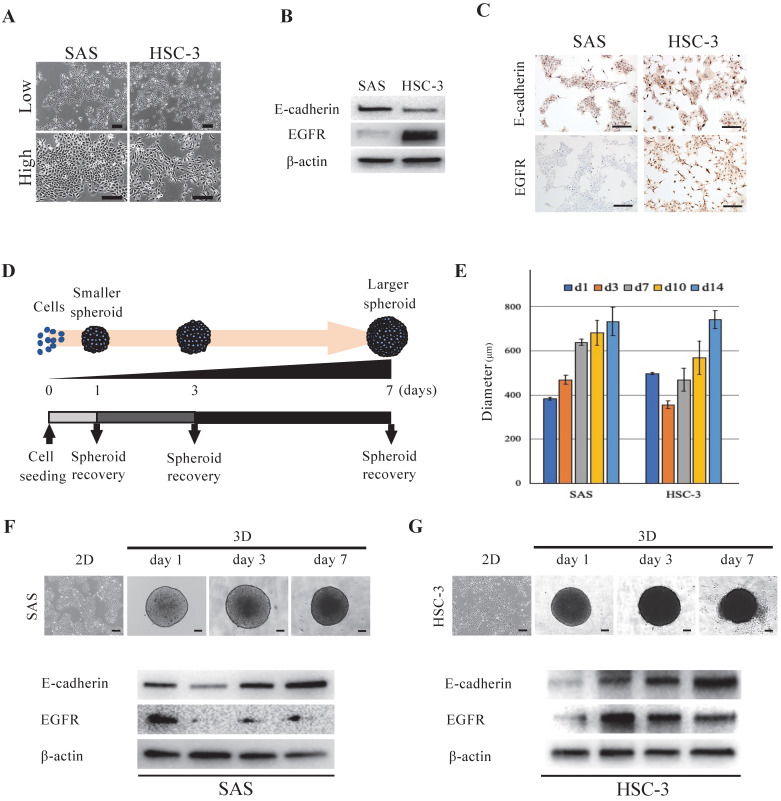
** Morphology of oral cancer cells in 2D and 3D culture. (A)** Representative images of SAS and HSC-3 cells in 2D culture. Upper, low magnification images. Bottom, high magnification images. Scale bars, 200 µm. **(B)** WB showing the levels of E-cadherin and EGFR in SAS and HSC-3 cells. **(C)** IHC images showing positive staining for E-cadherin and EGFR in SAS and HSC-3 cells. Scale bars, 200 µm. **(D)** Schematic of the experimental protocol for achieving spheroid recovery over time. **(E)** Comparison of spheroid diameter changes over time. The experiment was performed in five independent culture wells. **(F, G)** Morphology and protein expression of oral cancer cells cultured as 3D spheroids in ULA 96-well plates. Scale bars, 200 µm. The WB results show the levels of E-cadherin and EGFR in (F) SAS cells and (G) HSC-3 cells.

We used two oral cancer cell lines, SAS and HSC-3, that differed with respect to their cell morphologies, proliferation style and rate. The developmental dynamics of each cell line in monolayer culture were diverse, and the cell morphology varied from polygonal to paracircular to spindle-shaped (Fig. 1A). In order to further characterize each oral cancer cell line and examine whether differences in the expression of the cell adhesion molecule E-cadherin and epidermal growth factor receptor (EGFR) between cell lines affect the 3D spheroid growth and drug response, we first evaluated the cellular expression levels of E-cadherin and EGFR in 2D culture. Both oral cancer cell lines showed expression of E-cadherin, whereas EGFR expression was significantly higher in HSC-3 than in SAS cells (Fig. 1B). IHC staining of cultured cells showed similar levels of E-cadherin-positivity in both cell lines, but the level of EGFR-positivity was significantly higher in HSC-3 cells, which was similar to the WB results (Fig. 1C). Next, in order to examine whether the degrees of differentiation and malignancy affect the morphology and structure of spheroids, we transferred four oral cancer cell lines with different cell morphologies, colony formations and degrees of differentiation, i.e., SAS, HSC-3, HSC-4 and OSC-19, from 2D culture to 3D culture (Fig. 1D, S1, S2A) (Table 1) 12-14. Specifically, the adhered cells in 2D culture were stripped and seeded into plates for 3D culture, and daily morphological observations of tumor masses (spheroids) were performed and collected as needed (Fig. 1D). In 3D culture, a single spherical mass was formed from the day after seeding, and the spheroids of all cell lines increased in size and showed distinct shading over time (Fig. 1E-G, S2B). However, while the sizes of all spheroids increased across time, the spheroids of SAS, HSC-3, and OSC-19 grew in a monolocular form, while HSC-4 spheroids grew in a multilocular form (Fig. S2B). The maximal major diameter of these spheroids 2 weeks after seeding was approximately 600-800 μm for each of these cell lines (Fig. 1E). Spheroids of SAS and HSC-3 were collected on days 1, 3, and 7 after seeding, and WB showed temporal enhancement of E-cadherin (Fig. 1F,G). Interestingly, SAS showed a trend of attenuated EGFR expression in 3D culture compared to 2D culture, while conversely, HSC-3 showed a trend of enhanced EGFR expression in 3D culture compared to 2D culture (Fig. 1F,G). Incidentally, we similarly examined changes in E-cadherin and EGFR expression over time in 2D cultures, but the changes were not as clear as in 3D cultures (Fig. S3).

These data showed that forcing oral cancer cell lines with different characteristics into a 3D-culture environment resulted in a successively enhanced intercellular adhesion in both cell lines, while tumor growth-related molecules such as EGFR showed cell-specific differences in their increased or decreased expression.

### Pathological evaluation of oral cancer spheroids

**Figure 2:**
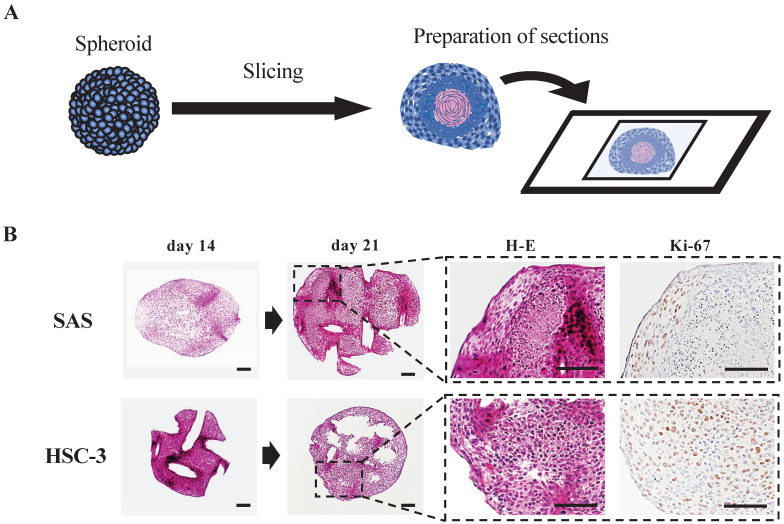
** Preparation and evaluation of oral cancer spheroid sections. (A)** Schematic of the experimental protocol for preparation of spheroid sections. **(B)** Representative H-E staining images and IHC staining images showing Ki-67 in SAS and HSC-3 spheroids. Scale bar, 100 µm.

To clarify the histology of the recovered spheroids, we embedded spheroids at each time, sectioned the paraffin-embedded blocks, stained the sections with H-E and DAB staining targeting specific proteins, and observed them using a microscope (Fig. 2, S2C, S4). The spheroids were growing in a spherical shape in culture, but H-E staining revealed a remarkable luminal structure in all oral cancer spheroids (Fig. 2B, S2C). In addition, SAS spheroids had sparse cell density in the center and high density in the edges from the early stage of 3D culture, and with time, the center showed necrotic tissue-like structures (Fig. 2B, S4A). The cells constituting the spheroid showed pathological characteristics peculiar to each cell line. For example, in the spheroid of HSC-4, which is a well-differentiated oral cancer cell line, small basal cell-like cells with poor cytoplasm were arranged at the margin, whereas cells with rich cytoplasm and a tendency to keratinize were gathered in the center (Fig. S4B). This feature was consistent with the general pathological findings of well-differentiated oral cancer. On the other hand, in the spheroid of SAS, the majority of cells were small cells, and a large necrotic area was observed in the center (Fig. S4B). This feature was consistent with the pathological findings of highly malignant poorly differentiated oral cancer. Immunostaining using the cell proliferation marker Ki-67 revealed a large number of Ki-67-positive cells at each spheroid margin (Fig. 2B, S2C).

These data suggested that the structure of spheroids made from oral cancer cells is likely to reflect the differentiation level and characteristics of the derived cancer cells.

### 3D culture is more clinically reflective of drug responsiveness than 2D culture

**Figure 3:**
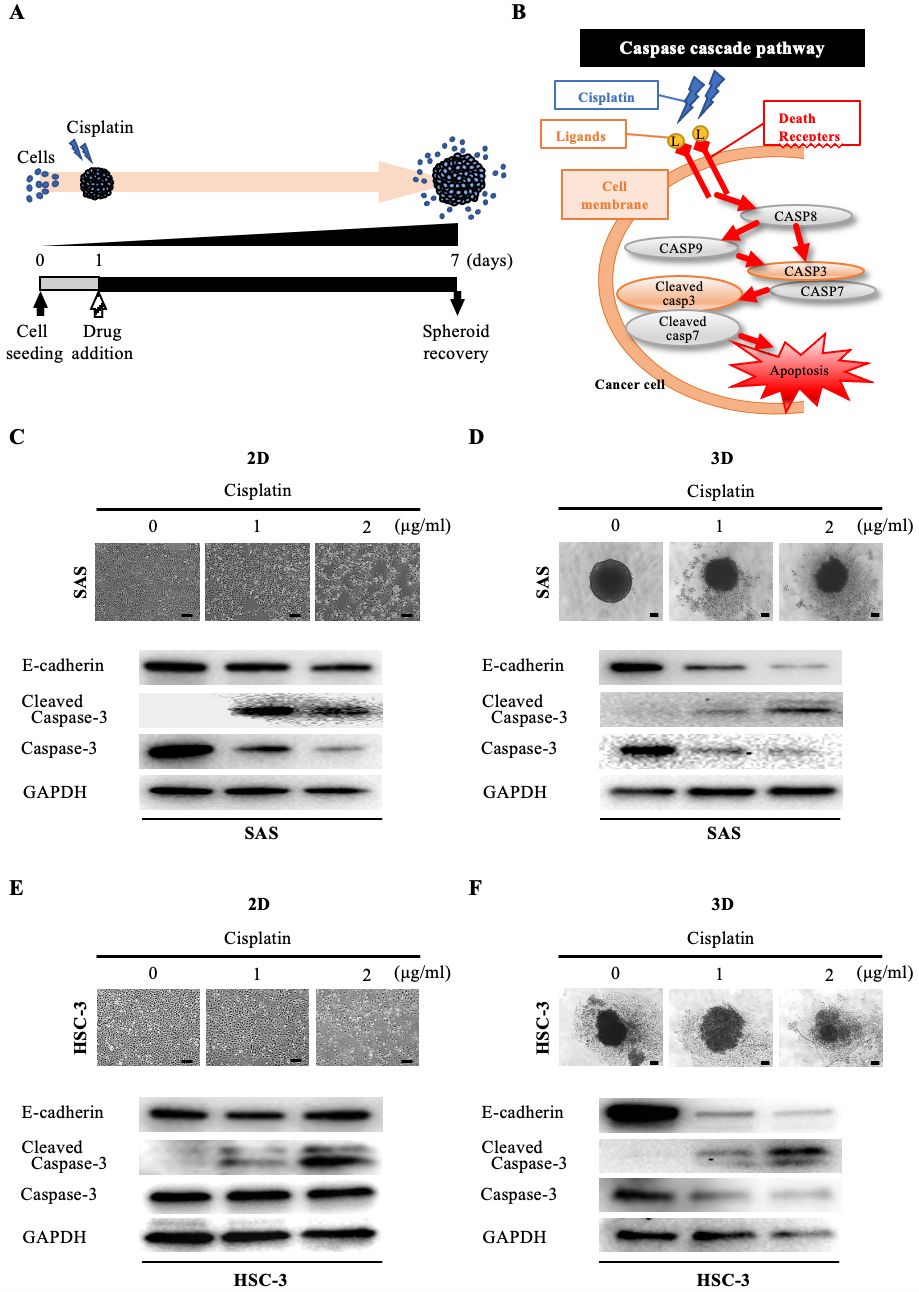
** Anti-tumor effect of cisplatin on oral cancer spheroids. (A)** Schematic diagram from cisplatin addition to spheroid recovery. **(B)** Schematic of the caspase cascade pathway during cisplatin-induced apoptosis. **(C-F)** Representative images of cells and changes in expression of E-cadherin and apoptosis-related molecules over 6 days of exposure to different concentrations of cisplatin. Scale bar, 200 µm. (C) SAS cells in 2D culture. (D) SAS cells in 3D culture. (E) HSC-3 cells in 2D culture. (F) HSC-3 cells in 3D culture.

We then assessed the morphological and molecular changes in the spheroid upon exposure to anticancer drugs. First, we evaluated the effect of cisplatin, which is widely used in oral cancer (Fig. 3A). Cisplatin has a cell-killing effect on cancer cells by inducing activation of the caspase pathway, which is a signaling pathway for apoptosis 15. In particular, cisplatin activates DNase and induces apoptosis by promoting cleavage and activation of caspase-3 and caspase-7 (increase in cleaved caspase) (Fig. 3B). Interestingly, when we exposed oral cancer cells to the IC50 concentration of cisplatin determined in 2D culture and compared their viability after 24 hours under 2D and 3D culture conditions, we found that the cell killing effect of cisplatin was predominantly downregulated under 3D culture conditions in both cell lines (Fig. S5). When we exposed SAS cells to graded concentrations of cisplatin under 2D-culture conditions, the cell density became increasingly sparse in a concentration-dependent manner (Fig. 3C). However, the WB data did not show any variation in E-cadherin, while it did show attenuation of caspase-3 and enhanced expression of cleaved caspase-3, with both effects being cisplatin-concentration dependent (Fig. 3C). On the other hand, when we exposed SAS cells to cisplatin under 3D-culture conditions, we observed a concentration-dependent disintegration of the spheroid, as well as attenuation of E-cadherin (Fig. 3D). In addition, there was a concentration-dependent attenuation of caspase-3 and increased expression of cleaved caspase-3 (Fig. 3D). Similarly, we compared the response of HSC-3 cells to cisplatin in 2D and 3D cultures (Fig. 3E,F). We observed a concentration-dependent decrease in cell density and non-variation of E-cadherin in HSC-3 under 2D cultures, and a concentration-dependent disintegration of spheroids and attenuation of E-cadherin in HSC-3 under 3D cultures, which were the same results as observed for SAS cells (Fig. 3E,F). We also obtained similar results for the concentration-dependent enhancement of cleaved caspase-3 expression, but notably, we did not observe any variation of caspase-3 in HSC-3 under 2D culture (Fig. 3E,F).

These data suggested that the cytotoxic, apoptosis-inducing effect of cisplatin was characterized by spheroid disintegration associated with activation of caspase-3 and attenuation of E-cadherin.

**Figure 4:**
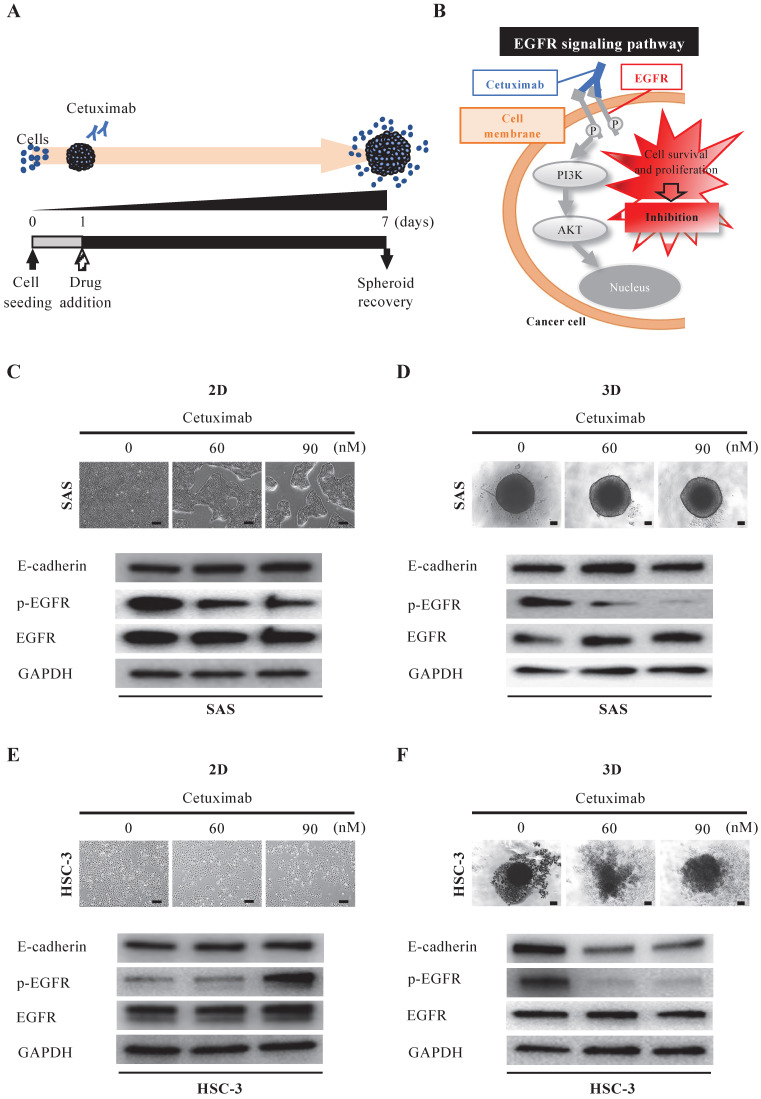
** Anti-tumor effect of cetuximab on oral cancer spheroids. (A)** Schematic diagram from cetuximab addition to spheroid recovery. **(B)** Schematic of the inhibition of the EGFR downstream signaling pathway by cetuximab. **(C-F)** Representative images of cells and changes in expression of E-cadherin, p-EGFR and EGFR over 6 days of exposure to different concentrations of cetuximab. Scale bar, 200 µm. (C) SAS cells in 2D culture. (D) SAS cells in 3D culture. (E) HSC-3 cells in 2D culture. (F) HSC-3 cells in 3D culture.

Next, we evaluated the efficacy of cetuximab, the only molecularly targeted drug available for oral cancer (Fig. 4A). Cetuximab exerts its antitumor effect by inhibiting the function of EGFR on the cell membrane and blocking cell proliferation signaling (Fig. 4B). When we exposed SAS cells to cetuximab under 2D-culture conditions at graded concentrations, cells formed island-like cell aggregates in a concentration-dependent manner (Fig. 4C). However, the WB data showed no variation in EGFR and its downstream molecules, let alone E-cadherin (Fig. 4C). On the other hand, when we exposed SAS cells to cetuximab under 3D-culture conditions, we observed neither concentration-dependent disintegration of the spheroid nor a concentration-dependent attenuation of E-cadherin, but the WB data showed a concentration-dependent inhibition of EGFR phosphorylation (Fig. 4D). We then compared the response of HSC-3 cells to cetuximab in 2D and 3D cultures in a similar manner (Fig. 4E,F). When we exposed HSC-3 cells to cetuximab under 2D-culture conditions at graded concentrations, the density of the cells became increasingly sparse in a concentration-dependent manner (Fig. 4E). However, the WB data showed a cetuximab concentration-dependent promotion of EGFR phosphorylation in addition to a constant level of E-cadherin (Fig. 4E). On the other hand, when we exposed HSC-3 cells to cetuximab under 3D-culture conditions, we observed a concentration-dependent inhibition of EGFR phosphorylation, and both of concentration-dependent disintegration of the spheroid and a concentration-dependent attenuation of E-cadherin (Fig. 4F).

These data suggested that the EGFR expression-dependent antitumor effects of cetuximab were more prominently reproduced in 3D culture and were characterized by spheroid disintegration in association with attenuation of E-cadherin.

### Immunohistological drug responsiveness evaluation of oral cancer spheroids

**Figure 5:**
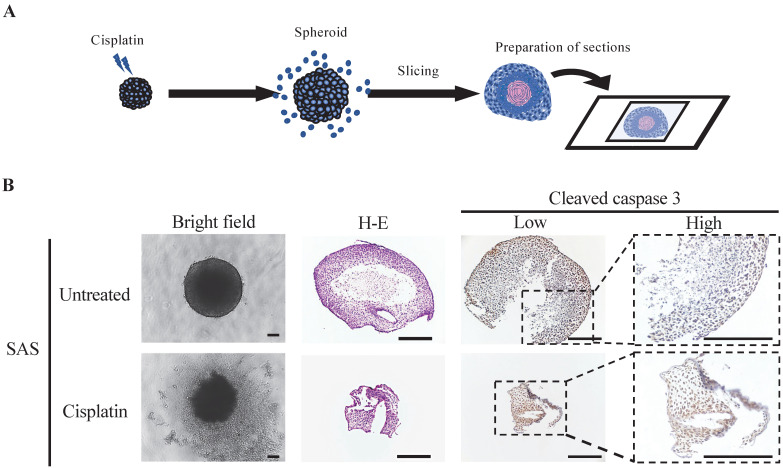
** Spheroids as a tool for determining drug efficacy. (A)** Schematic of the experimental protocol for preparation of drug-stimulated spheroid sections. **(B)** Representative bright field images, H-E staining images and IHC staining images showing cleaved caspase-3 in SAS spheroids with or without cisplatin for 6 days. Scale bar, 200 µm.

Finally, we examined the molecular changes resulting from the exposure of oral cancer spheroids to the anticancer drugs (Fig. 5). SAS spheroids subjected to the anticancer effect of cisplatin were collected and sectioned for various stainings (Fig. 5A). H-E staining showed a circular cross-sectional image with necrotic tissue in the center in spheroids without cisplatin, while the cross-sectional image was distorted in spheroids with cisplatin (Fig. 5B). IHC staining with cleaved caspase-3 showed a clear difference in positivity: spheroids without cisplatin tended to show positive cells at the margins, whereas spheroids with cisplatin showed positive cells throughout the entire area (Fig. 5B).

These data suggested that the drug responsiveness of spheroids could be evaluated histologically, and demonstrated the utility of oral cancer spheroids as a drug screening model.

## Discussion

### Usefulness of the spheroid model in 3D culture

There is a need to develop more effective treatments for many types of cancer. However, it is a major challenge to recreate the complex TME *in vitro*. As a result, although many drugs have shown good results *in vitro*, most have failed to transfer to *in vivo* models, and only a few of the newly approved anticancer drugs have shown antitumor activity in clinical trials 16. In other words, it is clear that anticancer drug screening on a platform using 2D-cultured cell lines cannot accurately select clinically effective compounds.

In cancer research, the benefits of using 3D models for* in vitro* studies have been well reported over the past few years 17-19. 3D cell culture may better mimic the TME *in vivo* and may help bridge the gap between 2D cell culture *in vitro* and living tissue *in vivo*, such as by maintaining the native cell phenotype and function 20-22. In other words, it has been suggested that spheroid models could be used as a tool for preclinical research in place of *in vivo* models, including mouse models for drug screening 17, 23. It is well known that 3D culture environments alter many cellular phenotypic and functional activities, such as proliferation, migration, viability, differentiation, drug sensitivity, and hypoxia, compared to 2D cultures 17, 24-27. Cancer cell-derived organoid models of prostate, lung, and pancreatic cancers have also been established as *in vitro* systems to model tumor pathogenesis *in vivo*, including tumor-associated signaling pathways and chemotherapy resistance 28, 29. In this study, we created a tumor tissue-like model by 3D culture using several types of oral cancer cell lines, and evaluated its tumor histology and drug responsiveness.

### Intercellular adhesion proteins expression and spheroid formation

The role of intercellular adhesion proteins in tumor progression, invasion and metastasis, and treatment resistance is one of the most closely studied issues in cancer biology. Intercellular adhesion proteins are considered to be participants in the regulatory signaling cascade associated with cell proliferation 30. Decreased amounts of intercellular adhesion proteins or loss of their function can be considered as a sign of malignant transformation acquisition 31. We previously reported that extracellular vesicles (EVs) derived from a highly metastatic oral cancer cell line promoted malignant transformation by acting on the loss of intercellular adhesion protein function of an immortalized oral mucosal epithelial cell line 32. At the same time, however, the data on the involvement of adhesion proteins in carcinogenesis and tumor progression are conflicting. The prevailing view is that adhesion proteins are tumor suppressors, and their high expression in tumor cells is associated with an epithelial phenotype (i.e., reduced invasiveness), while their loss is associated with increased epithelial-mesenchymal transition (EMT), proliferative activity, and migration 32-36. However, several reports have clearly demonstrated that overexpression of adhesion proteins also leads to malignant transformation. For example, in a variety of cancers, increased expression of desmoglein-2 has been shown to be associated with increased tumor grade 37-41.

Schmidt et al. reported that spheroid morphology in head and neck cancer is associated with E-cadherin and Ki67 expression 42. E-cadherin is expressed on the cell surface of most epithelial tissues. The extracellular region of E-cadherin supports calcium-dependent trans-homophilic binding and provides specific interaction with neighboring cells. E-cadherin is a key molecule in the maintenance of epithelial integrity, is involved in the regulatory mechanisms of epithelial cell proliferation, differentiation, and survival, and acts as a central regulator of processes involved in tumorigenesis and cancer progression, including invasion and metastasis 43. In our present experiments, the morphology of the spheroids cultured for 7 days differed greatly between the two oral cancer cell lines, but both showed temporal enhancement of E-cadherin (Fig. 1F,G). A similar situation has been previously reported in pancreatic adenocarcinoma cells and head and neck cancer cells, which exhibit stronger cohesion by binding cells together, ensuring tissue integrity during cell population migration 44-46. Loss of epithelial phenotype and intercellular adhesion due to decreased expression of E-cadherin, a transmembrane protein at the junction of adherent cells expressed on differentiated and polarized epithelial cells, is an important early event during EMT and is considered to be a prerequisite for cancer to progress to metastatic disease 47. However, tumors are heterogeneous, and cancer cells may retain certain epithelial properties, such as E-cadherin expression, to promote invasion. In fact, the loss of E-cadherin does not necessarily correlate with invasiveness 48. Meanwhile, several studies have reported elevated levels of E-cadherin in tumor spheroids 43, 49, 50. Xu et al. reported that E-cadherin expression is upregulated during spheroid formation in ovarian cancer cells compared to monolayer cells, and this change may contribute to cisplatin resistance 51. In spheroids of anaplastic thyroid carcinoma, it has been shown that the expression of E-cadherin is increased compared to that in monolayer cells while the expression of tight junction proteins is lost 49. In our oral cancer cell results, we observed a temporal enhancement of E-cadherin in spheroids (Fig. 1F,G).

### Histopathological evaluation of spheroids as cell aggregates

Cell lines exhibiting an epithelial phenotype have also been shown to form more regular and uniform cell aggregates 52. Eguchi et al. classified the types of cell aggregates formed by 67 cell lines cultured on 3D morphology-enhancing NanoCulture Plates (NCPs) and found that 49 cell lines formed spheroids of spherical shape, 8 cell lines formed grape-like aggregates, 8 cell lines formed other aggregates, and 3 cell lines formed monolayer sheets 17. One advantage of 3D culture is that it allows for histopathological evaluation of cell aggregates (Fig. 2). Arita et al. cultured spheroids of primary cancer cells from patient specimens for extramammary Paget's disease, for which cell lines have not yet been established, and used histopathological evaluation to determine drug efficacy, showing the possibility of selecting the most effective drug for each patient 53. Maru et al. performed 3D cultures of 21 gynecological tumors and examined whether the proliferated organoids retained various features of the original tumors by histopathological examination and targeted genome sequencing 54. As a result, they reported that *in vitro* analysis of the drug efficacy of paclitaxel and cisplatin were feasible using organoid-derived spheroids 54. Sections prepared from our oral cancer cell spheroids also showed some pathological findings characteristic of actual oral cancer tissues (Fig. 2, S4). For example, the spheroid of HSC-4, a highly differentiated oral cancer cell line, had an array of small basal cell-like cells with poor cytoplasm at the margins, while the center had a cluster of cells with rich cytoplasm and a tendency toward keratinization (Fig. S4). This is consistent with the general pathological findings of highly differentiated oral cancer. On the other hand, the spheroids of SAS and HSC-3 showed extensive necrotic areas in the center with a majority of small cells. This is consistent with the findings of high-grade oral cancer with a tendency toward poor keratinization (Fig. 2, S4). Liao et al. also created spheroids using hepatocellular carcinoma (HCC) cells derived from two patients and performed immunohistochemical staining for ARG-1 (a highly sensitive and specific marker of HCC) using the primary tissue and spheroids 55. This study showed that the spheroids not only maintained the ARG-1 expression of the original tumor, but also showed differential expression according to the differentiation level of the original tumor. Furthermore, the morphology of the cells also resembled that of HCC cells *in vivo*, indicating that the HCC *in vivo* was well simulated by the 3D spheroid model 55. The analysis of *in vitro* spheroids, which have characteristics and histology more similar to those of real clinical tumor samples, has the potential to help overcome cancer.

### Spheroid culture as a tool for evaluating the efficacy of anticancer drugs

One of the objectives of this study was to compare the two culture systems in terms of the survival of oral cancer cells after treatment with cisplatin and cetuximab (Fig. 3-5). Previous studies have shown that the 3D shape of the spheroid may affect drug penetration and that these phenomena may explain the high resistance to chemotherapy treatment 56. Song et al. showed that the IC50 values for cisplatin and paclitaxel in ovarian cancer cell lines cultured in 3D on hydrogel, matrigel, and collagen I were significantly higher than those in 2D-cultured cells 16. In other cancer types, 3D-cultured tumor cells have also shown very high resistance to chemotherapeutic agents or reversal of the cell phenotype 57, 58. On the other hand, Kutova et al. showed that in ovarian cancer cell lines, the resistance of spheroids to the action of doxorubicin and HER2 inhibitors was comparable to that of monolayer cultures 59. They noted that the comparable responsiveness of monolayers and spheroids to doxorubicin treatment is consistent with the lower density of ovarian cancer spheroids and the absence of an “epithelial-like” cell layer. At the same time, the cell entry and subsequent toxic effects of HER2 inhibitors are strongly dependent on the presence of HER2 on the cell surface. Even among the two anticancer drugs we used in this study, there were differences in the results of action on oral cancer spheroids between the cytotoxic agent and the molecular target-based agent (Fig. 3B, 4B). Cisplatin, a DNA-damaging agent, induces apoptosis in cancer cells and exhibits anti-tumor effects. It is a well-known anticancer drug widely used in chemotherapy for various human cancers, including oral cancer 39, 60, 61. In our experiments, cisplatin treatment of 3D-cultured oral cancer cell spheroids resulted in concentration-dependent cell separation from the spheroids (tumor disintegration), attenuation of E-cadherin, and increased expression of cleaved caspase-3, an activator of the apoptosis-inducing factor caspase-3, in both cell lines (Fig. 3,5). Cetuximab, on the other hand, is highly dependent on the presence of EGFR on the cell surface, and shows anti-tumor effects by inhibiting its function and blocking cell growth signaling. When we treated 3D-cultured oral cancer cell spheroids with cetuximab, the SAS spheroids showed no difference in morphology and no change in E-cadherin among the cetuximab concentrations, whereas the HSC-3 spheroids showed a concentration-dependent disintegration of spheroids and attenuation of E-cadherin (Fig. 4). The finding that there was little tumor damage in SAS spheroids despite the inhibition of EGFR phosphorylation by cetuximab is probably attributable to the very low basal expression of EGFR in untreated SAS spheroids. Jedlinski et al. previously demonstrated that head and neck squamous cell carcinoma cell lines with lower EGFR expression than normal oral keratinocytes show increased proliferation rates after cetuximab exposure compared to untreated controls 62. Luca et al. reported that colorectal cancer cell lines in 3D culture showed decreased EGFR expression and increased resistance to the EGFR tyrosine kinase inhibitor AG1478 compared to 2D culture 63. We believe that the differences in the effects of these two drugs in this study may reflect the clinical significance of their respective antitumor properties.

### Future clinical applications of spheroid culture

It has been reported that tumor spheroids that appear identical in shape may exhibit differential sensitivity to drugs that cannot be explained by molecular or metabolic variables 64. Rather, specific characteristics of the cell or patient determine the outcome of the treatment. This suggests that in the absence of molecular predictors of chemotherapy drug selection, experimental drug screening using patient-derived cells should be used as an approach to personalized medicine 65. Our 3D-culture model used in this study is an accessible and economical tool that can be used for such purposes. Since it does not require advanced technology or equipment, it could be widely implemented in many medical institutions.

## Conclusion

**Figure 6:**
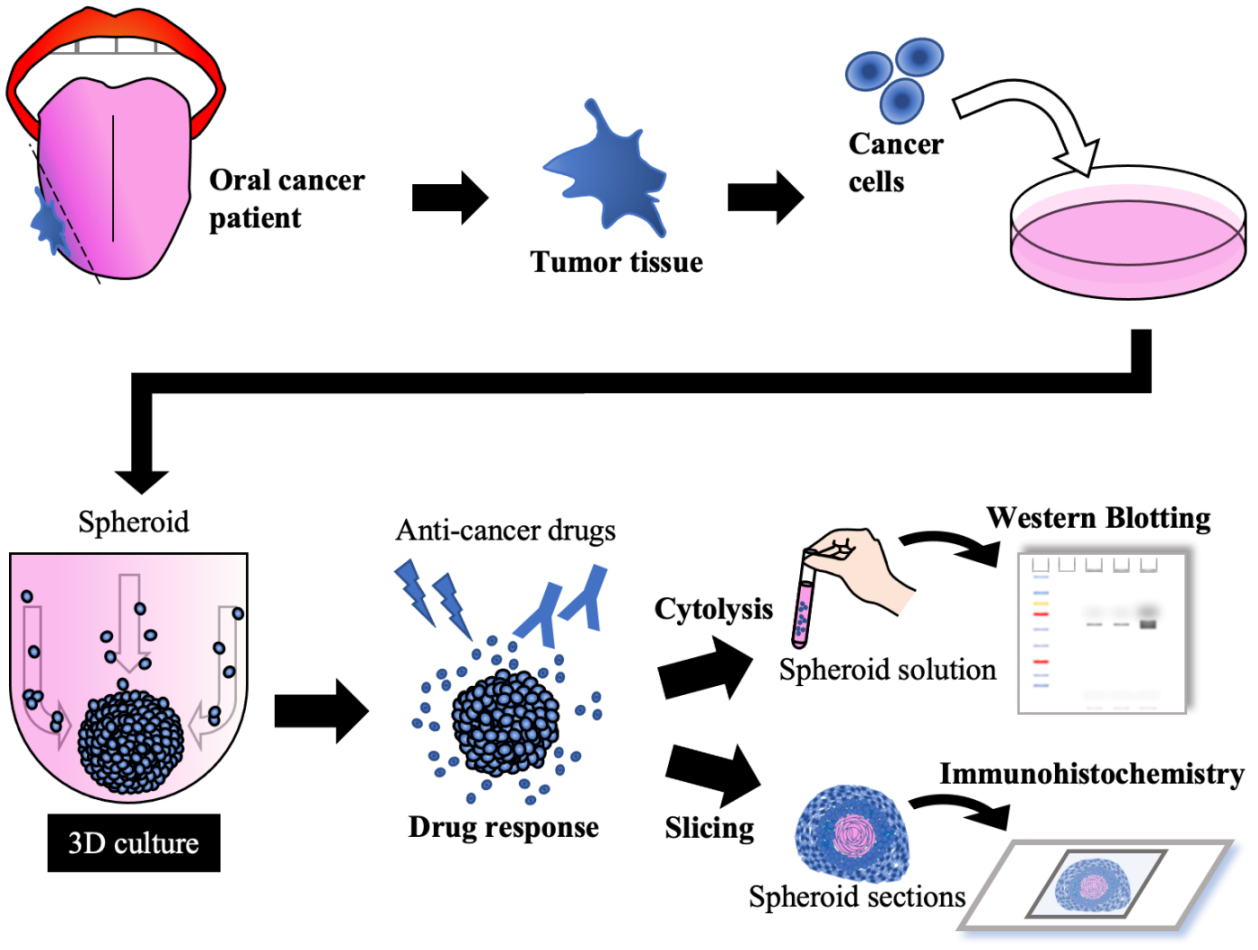
Future concepts using 3D cultures in oral cancer treatment.

In this study, we generated spheroids from oral cancer cell lines, and their structures appeared to have properties similar to the corresponding clinical tumor histology. Three-dimensional culture has been an important tool for developing assays to determine the drug chemosensitivity of cells as a platform for preclinical or clinical drug screening. In the future, spheroids/organoids could be generated from tissue samples obtained from patients with oral cancer and applied to drug screening and the development of individualized diagnosis and treatment while maintaining cancer-specific functionality (Fig. 6).

## Supplementary Material

Supplementary figures.

## Figures and Tables

**Table 1:** List of human oral cancer cell lines used in the experiments

Cell line	Ref.	Sex	Age	Primary site	Histological type	Sampling site	Cell morphology	Colony formation	Differentiation
SAS	12	F	69	tongue	SCC	primary tumor	polygonal, circular	islanded	poorly
HSC-3	13	M	64	tongue	SCC	metastatic LN	spindle-shaped	scattered	poorly
HSC-4	13	M	64	tongue	SCC	metastatic LN	polygonal	islanded	well
OSC-19	14	M	61	tongue	SCC	metastatic LN	spindle-shaped	islanded	well

F: female; M: male; SCC: squamous cell carcinoma; LN: lymph node.

## Abbreviations

2D: two-dimensional
TME: tumor microenvironment
3D: three-dimensional
ULA: ultra-low attachment
WCL: whole cell lysate
WB: western blotting
IHC: immunohistochemistry
H-E: hematoxylin and eosin
EGFR: epidermal growth factor receptor
EVs: extracellular vesicles
EMT: epithelial-mesenchymal transition
