# Supplementary material for: Reproduction of the Antitumor Effect of Cisplatin and Cetuximab Using a Three-dimensional Spheroid Model in Oral Cancer
Source: Int J Med Sci. 2022 Jul 18;19(8):1320–33. doi: 10.7150/ijms.74109 (PMC9346383; doi:10.7150/ijms.74109)

Figure S1. Representative images of oral cancer cells in 2D culture. Scale bar, 200 µm.

Figure S2. (A) Schematic diagram of the transition from 2D to 3D culture. (B) Representative time-specific images of oral cancer cells in 3D culture. Scale bar, 200 µm. (C) Representative H-E staining images and IHC staining images showing Ki-67 in HSC-4 and OSC-19 spheroids. Scale bar, 200 µm in the upper two rows, 100 µm in the lower two rows.

Figure S3. Morphology and protein expression of oral cancer cells in 2D culture. Scale bars, 200 µm. The WB results show the time-dependence of the levels of E-cadherin and EGFR in SAS cells and HSC-3 cells.

Figure S4. (A) Representative H-E staining images of SAS spheroids on culture days 3 (left) and 7 (right). Black lines indicate regions with sparse cell density: the central regions and necrotic regions. Red lines indicate regions with high cell density: the limbus and dense small cells (short internuclear distance). Blue lines indicated lumenal structures. Scale bar, 200 µm. (B) Representative H-E staining images in

two oral cancer spheroids with different differentiation. Poorly differentiated SAS spheroids had a necrotic region (left). Well-differentiated HSC-4 spheroids had an aggregation of keratinocytes (right). Scale bar, 100 µm.

Figure S5. (A) Representative images of oral cancer cells with or without cisplatin in 2D and 3D culture. Scale bar, 200 µm. (B) Cell survival rate of oral cancer cells under 2D and 3D culture in cisplatin at the IC50 concentration. $n = 3$. *$p<0.05$, **$p<0.01$ (paired t-test).

Supplementary figure 1

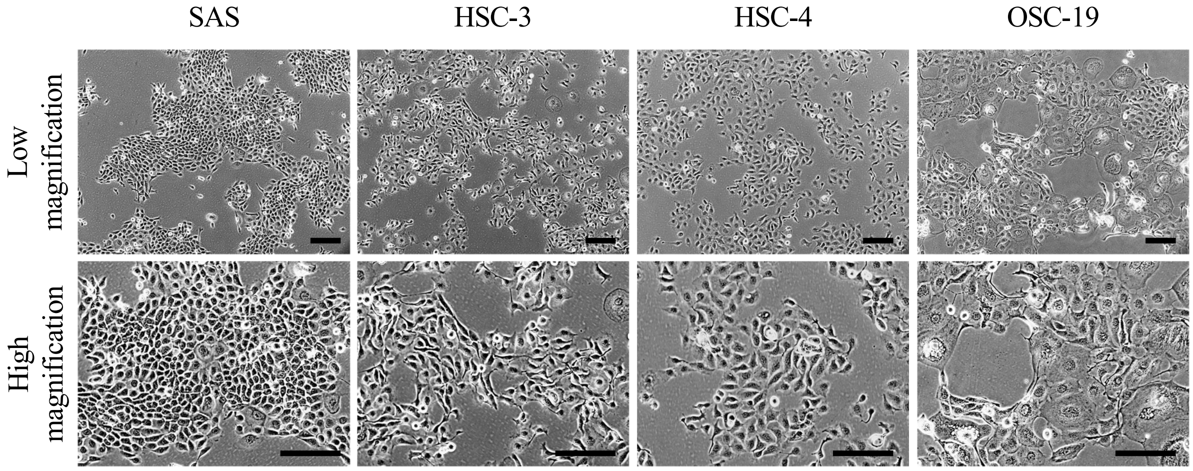

Supplementary figure 2

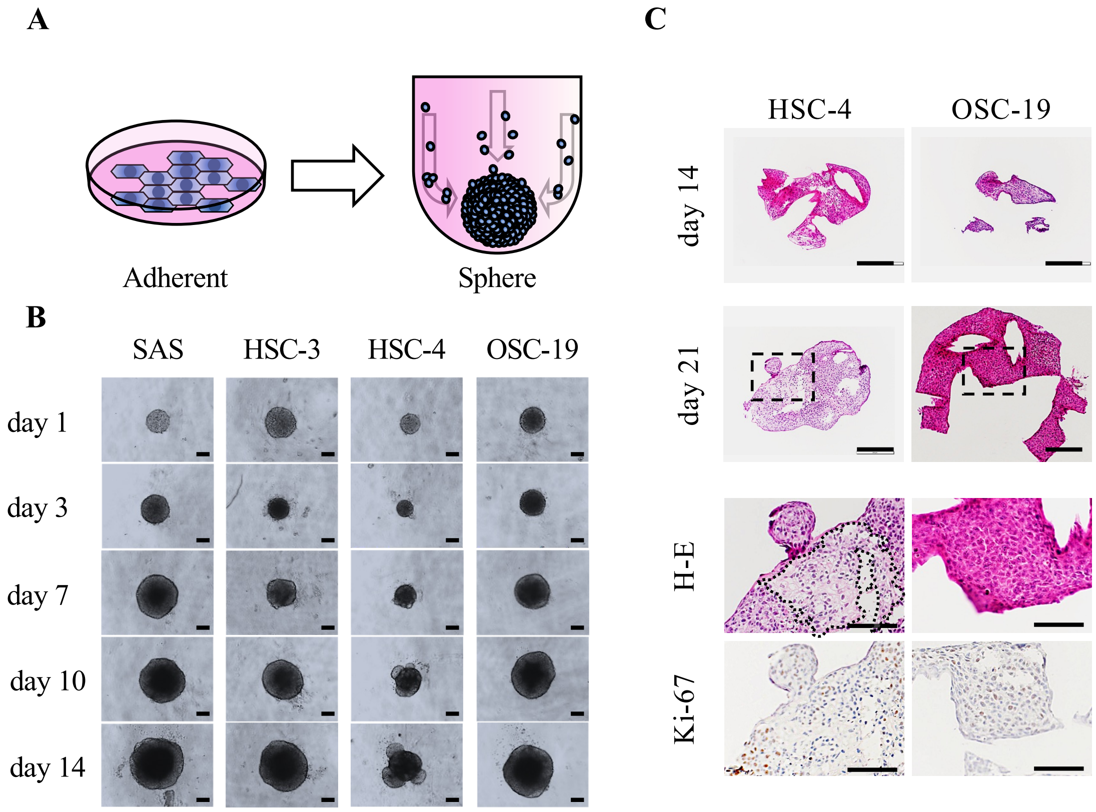

Supplementary figure 3

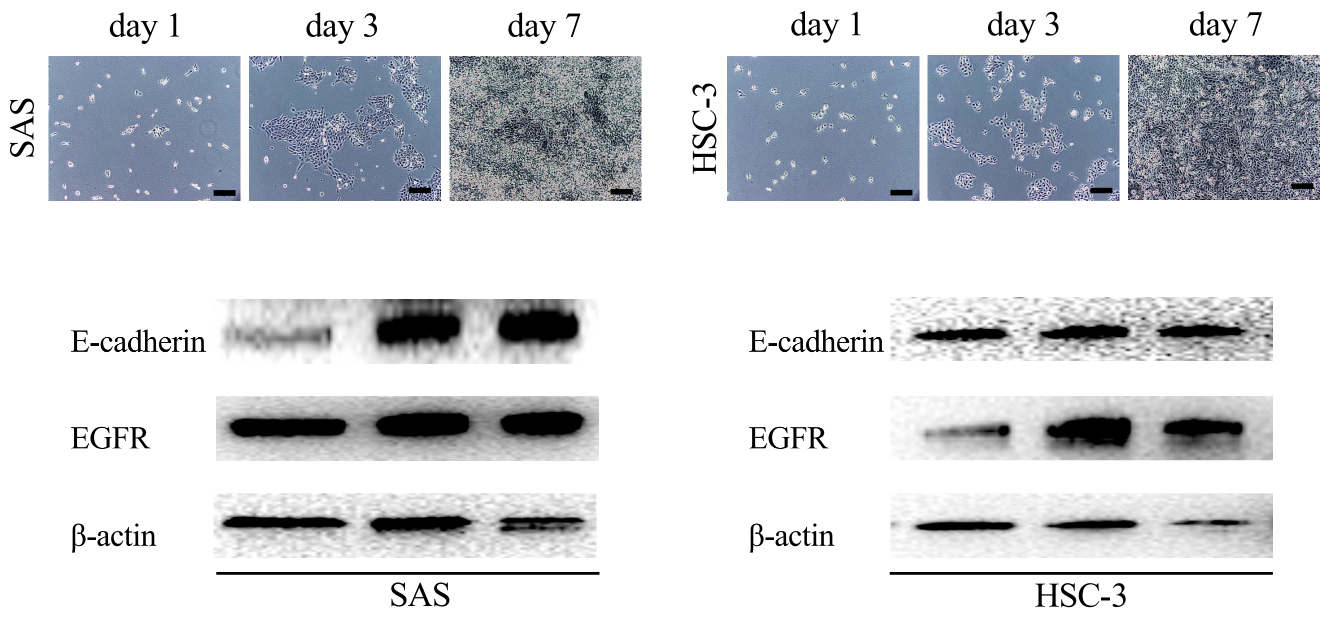

Supplementary figure 4

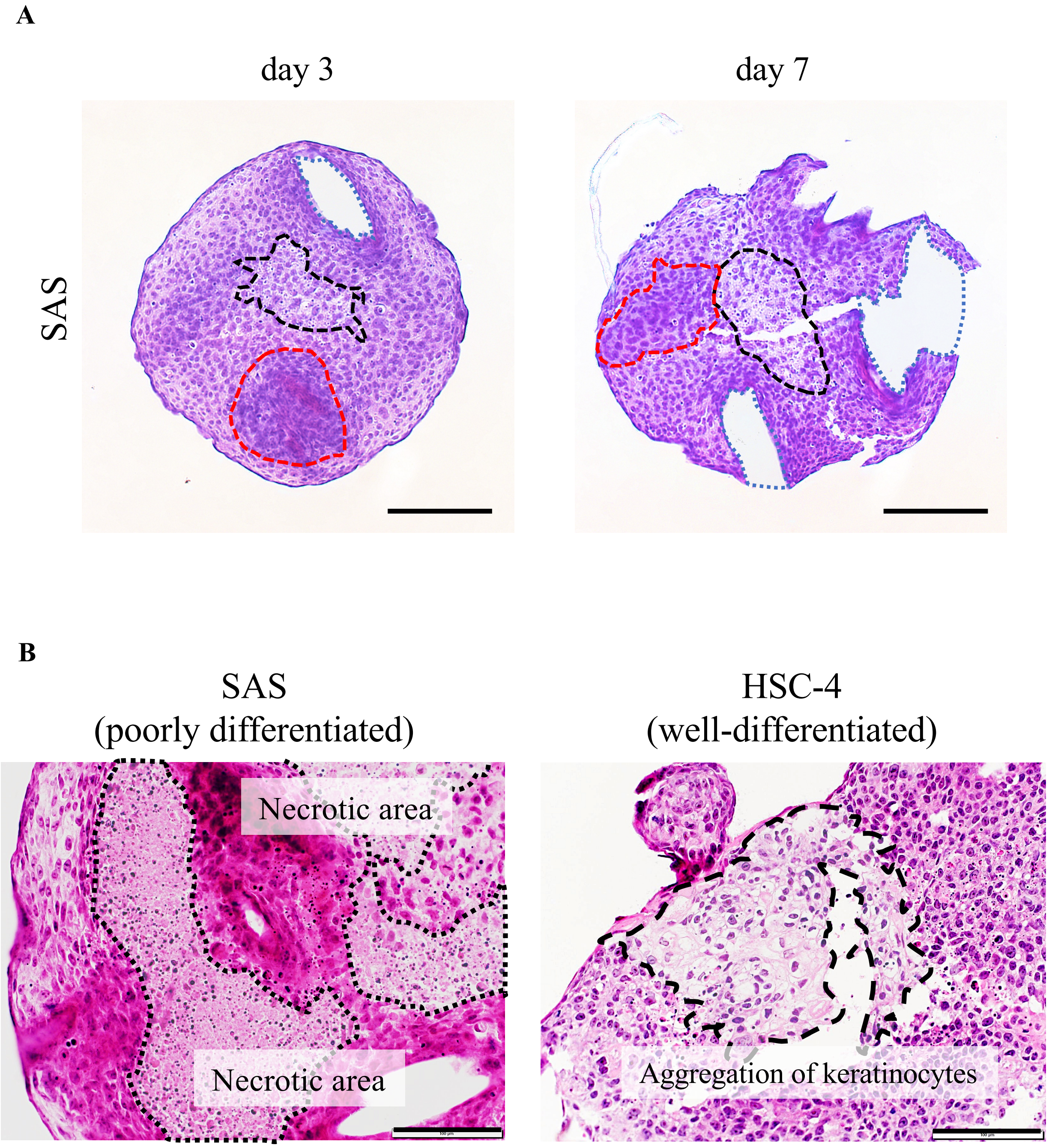

Supplementary figure 5

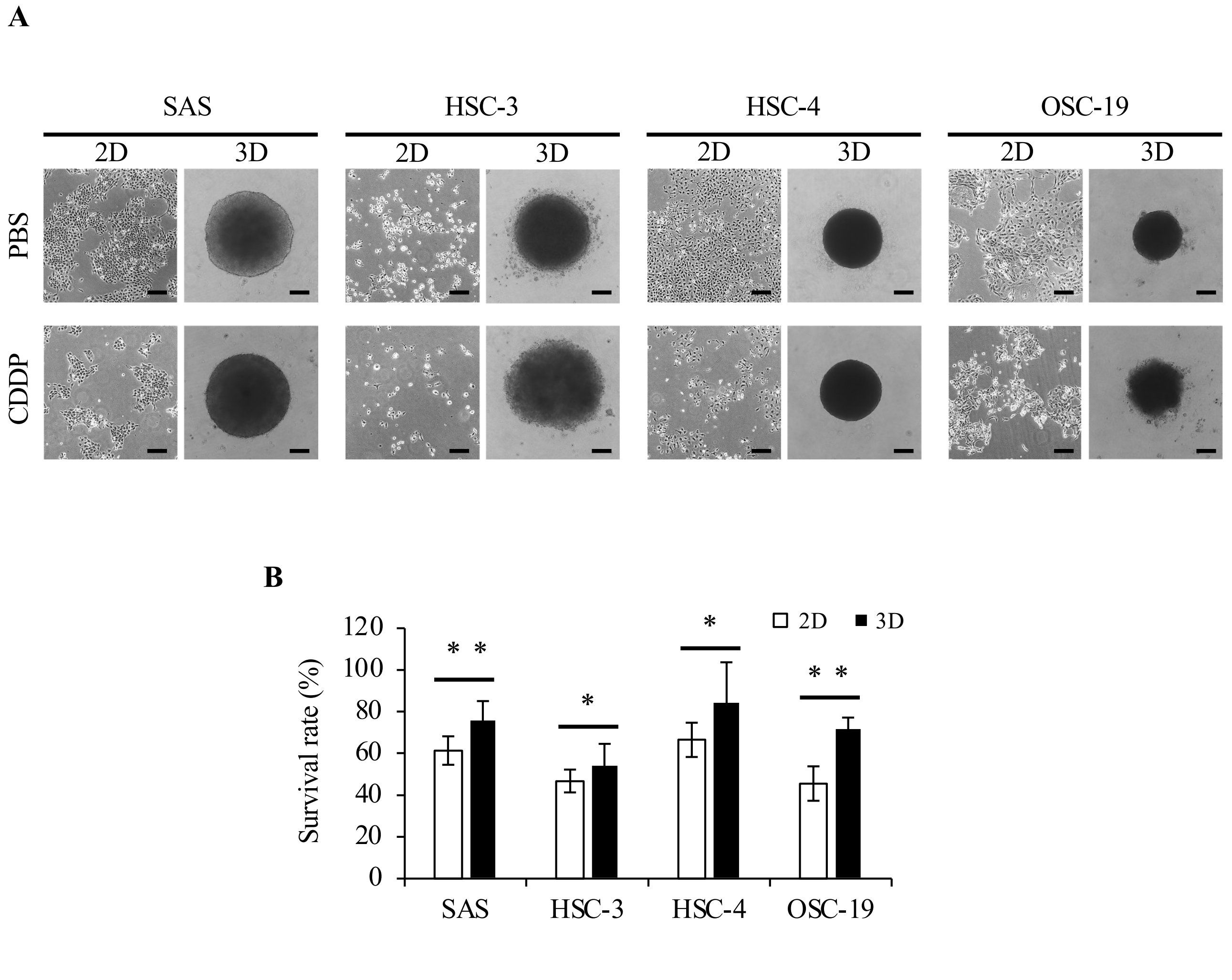

Supplement: Supplementary file 1 — Supplementary figures. [file ijmsv19p1320s1.pdf]
